# Disruption of key NADH-binding pocket residues of the *Mycobacterium tuberculosis* InhA affects DD-CoA binding ability

**DOI:** 10.1038/s41598-017-05042-4

**Published:** 2017-07-05

**Authors:** Daniel J. Shaw, Kirsty Robb, Beatrice V. Vetter, Madeline Tong, Virginie Molle, Neil T. Hunt, Paul A. Hoskisson

**Affiliations:** 1 Strathclyde Institute of Pharmacy and Biomedical Sciences, University of Strathclyde, 161 Cathedral Street, Glasgow, G4 0RE UK; 2 Department of Physics, University of Strathclyde, SUPA, 107 Rottenrow East, Glasgow, G4 0NG UK; 3 Laboratoire de Dynamique des Interactions Membranaires Normales et Pathologiques, Université de Montpellier II, Montpellier, France; 4 UCB, Slough, UK

## Abstract

Tuberculosis (TB) is a global health problem that affects over 10 million people. There is an urgent need to develop novel antimicrobial therapies to combat TB. To achieve this, a thorough understanding of key validated drug targets is required. The enoyl reductase InhA, responsible for synthesis of essential mycolic acids in the mycobacterial cell wall, is the target for the frontline anti-TB drug isoniazid. To better understand the activity of this protein a series of mutants, targeted to the NADH co-factor binding pocket were created. Residues P193 and W222 comprise a series of hydrophobic residues surrounding the cofactor binding site and mutation of both residues negatively affect InhA function. Construction of an M155A mutant of InhA results in increased affinity for NADH and DD-CoA turnover but with a reduction in V_max_ for DD-CoA, impairing overall activity. This suggests that NADH-binding geometry of InhA likely permits long-range interactions between residues in the NADH-binding pocket to facilitate substrate turnover in the DD-CoA binding region of the protein. Understanding the precise details of substrate binding and turnover in InhA and how this may affect protein-protein interactions may facilitate the development of improved inhibitors enabling the development of novel anti-TB drugs.

## Introduction

Tuberculosis (TB) remains a significant global health problem. An estimated 10.4 million people developed TB and 1.4 million died in 2015 as a result of infection with *Mycobacterium tuberculosis*, the causative agent of TB^1^. The TB problem is exacerbated by the emergence of multi-drug resistant (MDR) *M. tuberculosis*, of which there were an estimated 440,000 cases in 2008^2, 3^. There have been few major breakthroughs in TB treatment in the last 50 years^4^ and as a result there is an urgent need for novel drugs and treatment regimens to combat the spread of the disease.

Mycolic acids are vital, major components of the mycobacterial cell wall and their biosynthesis is a key validated target for many frontline anti-tubercular drugs such as Isoniazid (INH), ethionamide and Isoxyl^5, 6^. Mycolic acids have also recently been the focus of the development of new anti-tuberculosis drugs such as pyridomycin^7^. Mycolic acids are long chain α-alkyl β-hydroxyl fatty acids existing in two main forms in the mycobacterial cell; either as unanchored trehalose or glycerol esters, or as anchored mycolic acids, which are linked to the arabinogalactan component of the cell wall, contributing to the unique overall cell wall architecture of the mycobacteria^8^. In *M. tuberculosis*, mycolic acids are biosynthesised through the action of two fatty acid synthases, a eukaryotic-like type I and a prokaryotic-like type II enzyme termed FAS I and FAS II respectively. FAS I performs the *de novo* biosynthesis of C_16_ and C_24–26_ acyl-CoAs^9^. These medium-length acyl-CoAs then serve as primers for the FAS II system, being iteratively condensed through the action of the β-ketoacid-AcpM synthase III (FabH) with the malonyl-Acyl carrier protein (AcpM)^10, 11^. The second stage of elongation requires the action of the NADPH-dependent-β-ketoacyl-ACP reductase, MabA, resulting in the formation of β-hydroxyacyl-AcpM. The β-hydroxyacyl-AcpM is subsequently dehydrated by the HadABC dehydratases^12^ and then subsequently reduced by the 2-trans-enoyl-AcpM reductase, InhA^13^. The successive steps of condensation resulting in the long chain meromycolyl-AcpMs (up toC_56_), which are the direct precursors of mature mycolic acid, are performed by the β-ketoacyl-AcpM synthases (KasA and KasB)^14, 15^.

A key enzymatic step in this pathway is the 2-trans-enoyl-AcpM reductase, InhA, which belongs to a family of short-chain reductases^13, 16^ (Pfam Family PF13561^17^) and is essential for growth and survival in mycobacteria^18^.

Recently it has been shown that InhA is controlled post-translationally by phosphorylation^19–21^, suggesting that there is a fine level control over mycolic acid biosynthesis and maturation in mycobacteria. Genetic inactivation of InhA results in the accumulation of the FAS I end product Hexacosanoic acid (C_26_) and is similar to that observed in INH treated cells^18^. INH is a pro-drug and *in vivo* there is a requirement for INH to be activated by the catalase peroxidase, KatG, resulting in the formation of a isonicotinoyl radical that reacts with NAD forming the InhA inhibitory INH-NAD adduct^22^. Interestingly much of the clinical resistance observed is due to recessive mutations in several genes (*katG, ndh, msh, nat*) whereas dominant mutations occur in *inhA* ^23^. InhA is the primary target of INH, ethionamide and triclosan^24, 25^, given the demonstration that the transfer of the *inhA* S94A mutant allele in *M. tuberculosis* is sufficient to confer resistance to both INH and ethionamide^2^. Moreover, over-expression of *inhA* also confers resistance to INH in mycobacteria^1, 26^.

InhA plays and essential role in the biosynthesis of mycolic acids in *M. tuberculosis* and remains one of our primary and validated anti-TB drug targets. Given the rise of MDR-TB in the clinic it is important that a full understanding of the catalytic mechanism of this enzyme is obtained. Specifically, the role played by all potential molecular contacts with substrate, cofactors and inhibitors and how these residues contribute to overall enzyme functionality will give us a better understanding of how to design more potent inhibitors of their activity. Here we examine previously unstudied catalytic pocket residues through mutations in terms of their kinetics relating to NADH and DD-CoA substrate binding and turnover. We demonstrate that mutation of NADH-binding pocket residues can have profound effects on DD-CoA binding and suggest that there are long range molecular interactions in the InhA protein.

## Results

Structural studies of InhA performed by Rozwarski *et al*.^3, 27^, suggest that hydrogen-bonding interactions within the NADH binding pocket of InhA are key to INH inhibitory activity. To investigate this, single point mutations of InhA were made that potentially disrupt key molecular interactions. The S94A mutation is known to confer INH resistance in the clinic and in experimental studies, with the direct role of this residue believed to be via coordination of a water molecule, within a wider hydrogen-bonding network^4, 27^. The work of Rozwarski *et al*.^5, 6, 27^, suggests that M155 interacts indirectly with the nicotinamide ring of NADH or the pyridine ring of the INH-NAD adduct via a coordinated water molecule. The hydrophobic residues P193 and W222 are part of a series of hydrophobic residues that surround the pyridine ring of the isonicotinic acyl group^7, 27^ (Fig. S1).

To establish the biochemical role of each of these residues, each site was mutagenized (Oligonucleotides are detailed in Table 1) to an alanine residue and the resulting mutant enzymes were assayed for biochemical function with the natural substrates NADH and DD-CoA and the ability to turnover NADH was tested in the presence of the drug adduct INH-NAD and compared to the WT InhA enzyme. All purified mutant proteins were subjected to Circular Dichroism (CD), to evidence correct folding of the mutant proteins. The CD spectra of WT and all mutant proteins exhibited spectra that could be superimposed on each other, indicating that changes to enzyme activity was not due to misfolding or major overall structural changes (data not shown).

**Table 1:** Site-directed mutagenesis oligonucleotides used to generate mutant InhA proteins. Underlined sequence indicates the altered codon within the oligonucleotide.

Allele	Oligonucleotide sequence
InhA	**Forward (F) -** 5′-cgacggggtggtgcatgcgattgggttca-3′
S94A	**Reverse (R) - 5′-**tgaacccaatcgcatgcaccaccccgtcg-3′
InhA	**F-** 5′-cccgagccgggcggcgccggcctacaac-3′
M155A	**R-** 5′-gttgtaggccggcgccgcccggctcggg-3′
InhA	**F-** 5′-gttgccgcaggcgctatccggacgc-3′
P193A	**R-** 5′-gcgtccggatagcgcctgcggcaac-3′
InhA	**F-** 5′-gctcgaggagggcgcggatcagcgcgct-3′
W222A	**R-** 5′-agcgcgctgatccgcgccctcctcgagc-3′

### Kinetic analysis of InhA and site-directed mutant proteins

**Figure 1:**
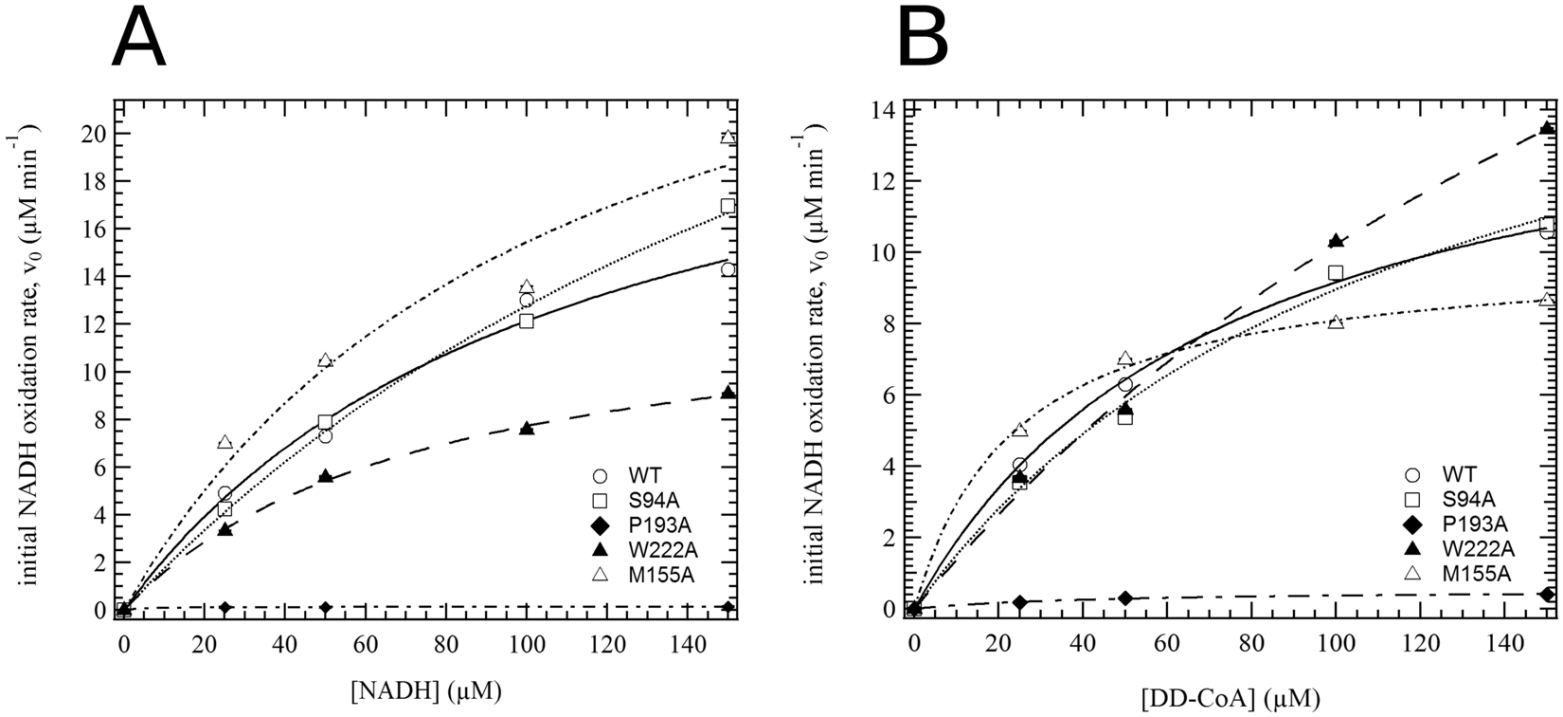
Enoyl reductase (ENR) activity of InhA and mutant derivatives. The enzymatic activity of the InhA variants and single point mutants were purified from recombinant *E. coli*, dialysed and assayed for ENR activity. (**A**) Enzymatic activity with increasing DD-CoA concentration (0–150 µM). Initial velocity for the WT and mutant enzymes measured at the NADH concentration of 100 µM. The lines are a fit to the data described by ${{\boldsymbol{v}}}_{0}=\frac{{{\boldsymbol{V}}}_{{\bf{\max }}}[{\bf{D}}{\bf{D}}-{\bf{C}}{\bf{o}}{\bf{A}}]}{{{\boldsymbol{k}}}_{{\boldsymbol{m}}}+[{\bf{D}}{\bf{D}}-{\bf{C}}{\bf{o}}{\bf{A}}]}\,$. (**B**) Enzymatic activity with increasing NADH concentration (0–150 µM). Initial velocity for the WT and mutant enzymes measured at the DD-CoA concentration of 50 µM. The lines are a fit to the data described by ${{\boldsymbol{v}}}_{0}=\frac{{{\boldsymbol{V}}}_{{\bf{\max }}}[{\bf{N}}{\bf{A}}{\bf{D}}{\bf{H}}]}{{{\boldsymbol{k}}}_{{\boldsymbol{m}}}+[{\bf{N}}{\bf{A}}{\bf{D}}{\bf{H}}]}$.

An overall kinetic analysis of WT InhA, InhA S94A, InhA M155A, InhA P193A and InhA W222A proteins is summarised in Tables 2 and 3 and Fig. 1. The WT and S94A InhA alleles exhibited similar values to those previously obtained in the literature and served as useful benchmark values with which to understand the role the additional residues that were studied here.

**Table 2:** NADH dependence (50 µM DD-CoA, 25–150 µM NADH varied).

Allele	Activity* (%)	K_m_[NADH] (μM)	V_max_[NADH] (µM min^−1^)	kcat (min^−1^)	kcat/K_m_[NADH] (μM^−1^ min^−1^)
WT	100 ± 4	98 ± 23	24.4 ± 5.4	1022 ± 224	10.4 ± 0.3
S94A	93 ± 6	211 ± 36	41.7 ± 6.9	1746 ± 291	8.5 ± 0.2
M155A	104 ± 6	65 ± 14	25.6 ± 4.6	1074 ± 193	16.6 ± 0.3
P193A	1 ± 2	5 ± 3	0.14 ± 0.01	5.8 ± 0.5	1.2 ± 0.6
W222A	58 ± 3	83 ± 7	14.7 ± 1.1	616 ± 45	7.5 ± 0.1

*The Activity column in this table is included to show a direct comparison of the initial turnover rates for each allele in the presence of fixed 50 µM DD-CoA and 100 µM NADH concentrations. The v0 of the wild-type turnover, v0 (WT), is defined as 100% activity, and is used to reference the other alleles. The final values are listed as percentage ratios of the v0 of each allele with respect to v0 (WT) generating the values listed.

**Table 3:** DD-CoA dependence (100 µM NADH, 25–150 µM DD-CoA varied).

Allele	K_m_[DD-CoA] (μM)	V_max_[DD-CoA] (µM min^−1^)	kcat (min^−1^)	kcat/K_m_[DD-CoA] (μM^−1^ min^−1^)
WT	75 ± 10	16.6 ± 0.8	694 ± 33	9.3 ± 0.7
S94A	106 ± 13	18.8 ± 1.8	789 ± 74	7.4 ± 1.2
M155A	26 ± 2	10.6 ± 0.5	446 ± 19	17.0 ± 1.5
P193A	53 ± 7	0.6 ± 0.1	25 ± 3	0.5 ± 0.1
W222A	145 ± 51	25.2 ± 8.6	1055 ± 359	7.3 ± 3.6

As previously shown^8, 16^, the S94A mutation has similar activity compared to WT but a reduced NADH affinity (K_m_), albeit with an increased V_max_ (Tables 2 and 3). Here we show that the affinity of InhA S94A for DD-CoA is also decreased, with a K_m_ of 106 µM compared to 75 µM for WT InhA, again however there is also an increase in V_max_ (Tables 2 and 3). The InhA S94A mutant kcat _NADH_ and kcat _DD-CoA_ was found to have increased 1746 min^−1^ and 789 min^−1^ respectively compared to 1022 min^−1^ and 694 min^−1^ for WT InhA. The kcat/K_m_ ratio for NADH indicates that the efficiency of the S94A mutants is 82% of the WT (Table 2: kcat/K_m_ of 8.5 μM^−1^ min^−1^ compared to 10.4 μM^−1^ min^−1^). The kcat/K_m_ ratio for DD-CoA indicates around 80% efficiency of the S94A mutant compared to the WT InhA.

The activity of an InhA M155A mutant was assessed to gain insight in to its effect on NADH and DD-CoA turnover. Interestingly the V_max_ for NADH was equivalent to that of WT, however the M155A mutants V_max_ for DD-CoA was around 64% of the WT rate (Fig. 1 and Tables 2 and 3). Overall the M155A mutant had an increased affinity (K_m_) for NADH (Table 2: 65 μM compared to 98 μM for WT). Moreover, modifying the M155 residue significantly increases the affinity of the mutant for DD-CoA (Table 3: 26 μM compared to 75 μM for WT). kcat of the InhA M155A mutant for NADH was similar to that of WT InhA (Table 2) however the kcat for DD-CoA was around 64% of the WT, suggesting that there may be long range structural interactions within the protein that are negatively affected by mutation of the M155 residue.

Mutation of P193 inactivates the ability of InhA to turn over either NADH and DD-CoA (Fig. 1A and B; Tables 2 and 3), which is consistent with the proposed role of P193 directly interacting with the nicotinamide group. The P193A mutant exhibits a much lower K_m_ for both NADH and DD-CoA, with residual activity of the mutant around 1% of the WT InhA. This also leads to much reduced catalytic efficiency of the InhA P193A mutant with an NADH kcat of 5.8 min^−1^ compared to 1022 min^−1^ for WT InhA, which indicates that this mutation is likely to be integral to the pocket geometry and function. The kcat/K_m_ ratio shows that the enzyme efficiency of InhA P193A with NADH is 12% of the WT and enzyme efficiency for DD-CoA is 5% of the WT. This suggests that this proline residue plays a key structural role in the distal area of the NADH binding pocket.

Similar to the role of residue P193, the tryptophan residue 222 is proposed to interact with the nicotinamide group^9, 27^. Kinetic analysis of the InhA W222A mutant indicates a reduction in overall enoyl reductase activity (Tables 2 and 3; Fig. 1). The W222A mutant showed an increased affinity (K_m_) for NADH but with a reduced V_max_ (Table 2), with a concomitant reduction in kcat when compared to WT InhA. The efficiency of the W222A mutant, when considering the kcat/K_m_ ratio shows that it is around 70% of the WT enzyme for NADH. Conversely the W222A mutation reduces the affinity of InhA for DD-CoA by around two-fold (Table 3), although V_max_ is increased along with the kcat for DD-CoA. The kcat/K_m_ ratio shows that the enzyme efficiency of an InhA W222A is around 80% of WT (Table 3). While it is proposed to have a similar interaction with NADH as P193^27^, W222 may play a role in the allosteric signalling to the DD-CoA binding region of the protein, or through mediating protein-protein interactions.

### Inhibition of InhA and mutants with INH-NAD

To test how the mutant derivatives of InhA behave when exposed to the inhibitory drug adduct, the rate of NADH turnover (V_max_) was measured when the proteins were incubated with 100 μM INH-NAD, prior to activity assays with the exception of InhA S94A, which showed no difference in activity from protein incubated in the absence of INH-NAD and was subsequently incubated with 300 μM INH-NAD to demonstrate inhibition (Table 4). These data indicate that inhibition of all mutants with INH-NAD is possible with residual activity following incubation with INH-NAD of 17% for WT InhA, 19% for InhA M155A, 23% for InhA W222A. Incubation of InhA P193A with INH-NAD showed a similar level of activity as the uninhibited protein, which was already shown to be inactive (Table 4).

**Table 4:** Activity and inhibition of InhA and mutant derivatives in the presence of INH-NAD.

Allele	V_max_ (μM min^−1^)
InhA WT	24.4 ± 5.4
InhA WT (100 µM INH-NAD)	4.2 ± 0.3 (17%)
InhA S94A	41.7 ± 6.0
InhA S94A (300 µM INH-NAD)	16.7 ± 1.7 (40%)
InhA M155A	25.6 ± 4.6
InhA M155A (100 µM INH-NAD)	4.9 ± 1.1 (19%)
InhA P193A	0.14 ± 0.01
InhA P193A (100 µM INH-NAD)	0.14 ± 0.01 (N/A)
InhA W222A	14.7 ± 1.1
InhA W222A (100 µM INH-NAD)	3.4 ± 0.2 (23%)

Concentration of adduct is indicated in brackets for inhibited reactions.

## Discussion

Previous studies of InhA have identified key residues that contribute to the mechanism of action of both the enzyme^10, 11, 28^, its activation through phosphorylation^12, 19^ and also contribute to our knowledge of the mechanism of action of the frontline anti-TB drug, INH. The results presented here expand our knowledge of key residues in the active site by adding information to our understanding that the role NADH turnover may play in potential allosteric mediated interactions with DD-CoA and/or the assembly of multimeric states of InhA. Solving the structure of InhA allowed the identification of residues within InhA responsible for binding NADH that may play a key role in binding both natural substrate and the inhibitory drug adduct INH-NAD^13, 22, 27^. The residues studied here were predicted to make significant molecular contacts with NADH or INH-NAD and are indicated in Fig. S1.

InhA mutations in clinical INH resistant strains are generally located in the NADH binding pocket^14–16, 29^, with a key residue being S94. This study of an S94A mutant confirmed previous data^12^ that this single residue change results in an increased turnover of the NADH substrate, via an increase in V_max_ coupled with a reduced affinity (K_m_) for NADH and the INH-NAD adduct, however this reduced affinity is not sufficient to explain drug resistance. The data presented here support this hypothesis, indicating that mutations resulting in altered geometry in the NADH binding pocket may have significant effects elsewhere in the protein, with the DD-CoA affinity of the S94A mutant also being altered despite the distance from this region of the protein. Moreover, the M155 and P193 mutations affect DD-CoA turnover and affinity in addition to the local effects observed in the NADH binding pocket. These observations fit with inter- and intramolecular signalling hypotheses of Rawat *et al*.^13, 16, 29^. and indicates that further work on interactions with the FASII complex and the molecular dynamics of the protein would be valuable in fully elucidating the mechanism of mycolic acid biosynthesis.

Work by Rawat *et al*.^17, 29^, also suggests that there is a two-step mechanism for InhA binding the INH-NAD adduct, where an initial weak binding of the adduct is followed by slow conversion to a tightly inhibited complex. Moreover, these authors suggest that protein-protein interaction, supported by the work of Bloch and others^18, 30, 31^, between multiple FASII pathway enzymes (including InhA) result in the formation of a functional mycolic acid biosynthetic complex. It is believed that these interactions may modulate the catalytic activity of InhA when a functional tetramer is formed and in a complex with other FASII enzymes gives rise to the appearance of allosteric binding characteristics of InhA kinetics^19–21, 29, 32^.

Intriguingly, an M155A mutation has an apparent decreased K_m_ for NADH and decreased K_m_ for DD-CoA suggesting an overall gain of function mutation via M155. These data show that mutation of a key NADH-binding pocket residue can have profound effects of other regions and activities of the enzyme. This is not simply mediated through binding of a substrate (NADH or INH-NAD) in the NADH-binding pocket as the InhA M155A mutant is inhibited by the INH-NAD drug adduct in a similar manner to WT InhA. These data suggest that the dynamics of catalytic activity and inhibition are subtler than previously thought and may point towards long range interactions within the molecule that are not easily observed through static crystallography approaches.

In summary, mutagenesis of key residues in the NADH-binding pocket of InhA can alter the enzymatic properties of both substrate binding pockets in a subtle manner and suggest that long-range intramolecular interactions may affect substrate turnover and binding for each of the substrates. Currently it is unclear how these mutations may affect assembly of the FASII protein complexes, but further work in these areas will allow deeper insight in to the inhibition of a fundamental cellular process for *M. tuberculosis* and may lead to novel therapeutics.

## Materials and Methods

### Bacterial strains and plasmids – *Escherichia coli*

DH5α (Invitrogen) was used for standard cloning/transformation and *E. coli* BL21 (DE3) Star (Novagen) was used for protein expression.

### Site-directed mutagenesis, expression and purification of recombinant InhA and mutant proteins

InhA was expressed from the plasmid pETPhos_*inhAWT* (N-terminal Histidine tagged). Site-Directed Mutants (SDMs) were constructed using the QuikChange II Site-Directed Mutagenesis kit (Agilent Technologies) according to the manufacturer’s instructions. SDM oligonucleotides are detailed in Table 1. All SDM plasmids were confirmed by sequencing. BL21 (DE3) Star freshly transformed with Wild-Type or mutant InhA plasmids were grown in LB medium with 100 μg ml^−1^ carbenicillin (Melford labs) overnight at 37 °C. These overnight cultures were used to inoculate 500 ml of LB and were grown under the same conditions, with shaking until they reach an A_600_ = 0.5. Isopropyl-thio-β-D-galactopyranoside (IPTG) was added to a final concentration of 1 mM and incubation was continued for 3 h at 37 °C. Cells were collected by centrifugation (4000 × *g*, 10 mins), washed and resuspended in Buffer A (20 mM Tris-HCl, pH 7.5, 500 mM NaCl, 10% Glycerol, and 20 mM imidazole) with Complete EDTA-Free Protease inhibitor cocktail (Roche) and Benzonase (Sigma-Aldrich). Cells were lysed using BugBuster (Novagen) according to the manufacturer’s instructions and lysates were clarified by centrifugation (4000 × *g*, 10 mins). InhA was purified by Ni^2+^-affinity chromatography (His-Trap, GE Healthcare) using an AKTA chromatography system. InhA was eluted from the column using Buffer B (20 mM Tris-HCl, pH 7.5, 500 mM NaCl, 10% Glycerol and 1 M imidazole). Fractions containing pure InhA (with the histidine-tag still intact, but has no effect on activity^18, 28^) were pooled, dialysed to remove imidazole and concentrated as required and stored in 20% glycerol at −20 °C as required. All data reported are the result of three technical replicates generated from three independent biological (independent over-expression) replicates.

### Assaying InhA enzymatic activity

The enoyl reductase activity of InhA was assayed spectrophotometrically following the oxidation of NADH to NAD^+^ at 340 nm. All measurements (unless stated) were performed using a Shimadzu UV-2550 dual-beam absorption spectrometer (Shimadzu Scientific Instruments). Reactions were carried out at 37 °C in 100 mM phosphate buffer, pH7.5 containing 1.47 µM of InhA (Wild-type or mutant protein) and varying levels of NADH and the *trans*−2-Dodecenoyl-Coenzyme A (DD-CoA) substrate. The data all activity measurements were single cuvette measurements run in triplicate over a total duration of 300 seconds. The DD-CoA substrate was synthesised from 2-Dodecenoic acid (Obtained from Enamine Ltd) using the anhydride method of Quémard *et al*.^13, 22^. The INH-NAD adduct was synthesised in the presence of InhA, producing inhibited InhA according to the method of Rozwarski *et al*.^22, 23^, where 350 μM WT or mutant InhA was incubated in 50 mM HEPES buffer (pH 7.5) with 17.5 mM NADH, 35 mM INH and 3.5 mM MnCl_2_. Inhibition assays for all alleles were conducted by removing aliquots from the incubation mixture previously described and spectrophotometrically monitoring the turnover of NADH to NAD^+^ at 340 nm in the presence of DD-CoA in the same manner as for the activity assays. All control reactions were collected simultaneously consisting of a fixed 100 µM concentration of NADH and the appropriate amount of DD-CoA but in the absence of enzyme. The kinetic parameters for all assays were determined at a fixed, saturating concentration of either NADH or DD-CoA (100 μM and 50 μM respectively), whilst varying the substrate or cofactor accordingly. Thus allowing the kinetics of inhibited WT InhA and mutant InhA to be determined under appropriate conditions. Values for K_m_ and V_max_ for each allele were calculated from a plot of a series of v_0_ values determined experimentally by varying one substrate concentration whilst the holding other at a fixed value. Values for K_m_ and V_max_ initially as a function of [NADH] and then [DD-CoA] are obtained directly by non-linear regression fitting of the Michaelis-Menten equation,

$${{\boldsymbol{v}}}_{0}=\frac{{{\boldsymbol{V}}}_{{\bf{\max }}}[{\bf{s}}{\bf{u}}{\bf{b}}{\bf{s}}{\bf{t}}{\bf{r}}{\bf{a}}{\bf{t}}{\bf{e}}]}{{{\boldsymbol{k}}}_{{\boldsymbol{m}}}+\,[{\bf{s}}{\bf{u}}{\bf{b}}{\bf{s}}{\bf{t}}{\bf{r}}{\bf{a}}{\bf{t}}{\bf{e}}]}$$

(where [substrate] represents [DD-CoA] or [NADH]) to the data. The equation was incorporated as a user-defined fitting function into the curve fitting analysis suite within the Igor Pro 6.37 [Wavemetrics] software which was used in all data/statistical analysis procedures. The fitting procedure utilises the Levenberg-Marquardt algorithm or Nonlinear Least-Squares method which returns estimates for K_m_ and V_max_ along with the standard error (±2σ) taken as the uncertainty.

## Electronic supplementary material

Supplementary Information
