# Supplementary material for: Disruption of key NADH-binding pocket residues of the Mycobacterium tuberculosis InhA affects DD-CoA binding ability
Source: Sci Rep. 2017 Jul 5;7:4714. doi: 10.1038/s41598-017-05042-4 (PMC5498604; doi:10.1038/s41598-017-05042-4)
Supplement: Supplementary file 1 — Supplementary Information [file 41598_2017_5042_MOESM1_ESM.doc]

**Disruption of key NADH-binding pocket residues of the *Mycobacterium tuberculosis* InhA affects DD-CoA binding ability**

**Daniel J. Shaw^1,2^,*^†^*, Kirsty Robb^1^, Beatrice V. Vetter ^1^, Madeline Tong^1^, Virginie Molle^3^, Neil T. Hunt^2^, and Paul A. Hoskisson^1*^**

**^1^**Strathclyde Institute of Pharmacy and Biomedical Sciences, University of Strathclyde, 161 Cathedral Street, Glasgow, G4 0RE, UK.

^2^Department of Physics, University of Strathclyde, SUPA, 107 Rottenrow East, Glasgow G4 0NG, UK.

^3^Laboratoire de Dynamique des Interactions Membranaires Normales et Pathologiques, Université de Montpellier II, France.

**Supplementary Fig. S1 -** PDB Entry 1ZID of InhA showing the locations of the residues mutagenised in this work


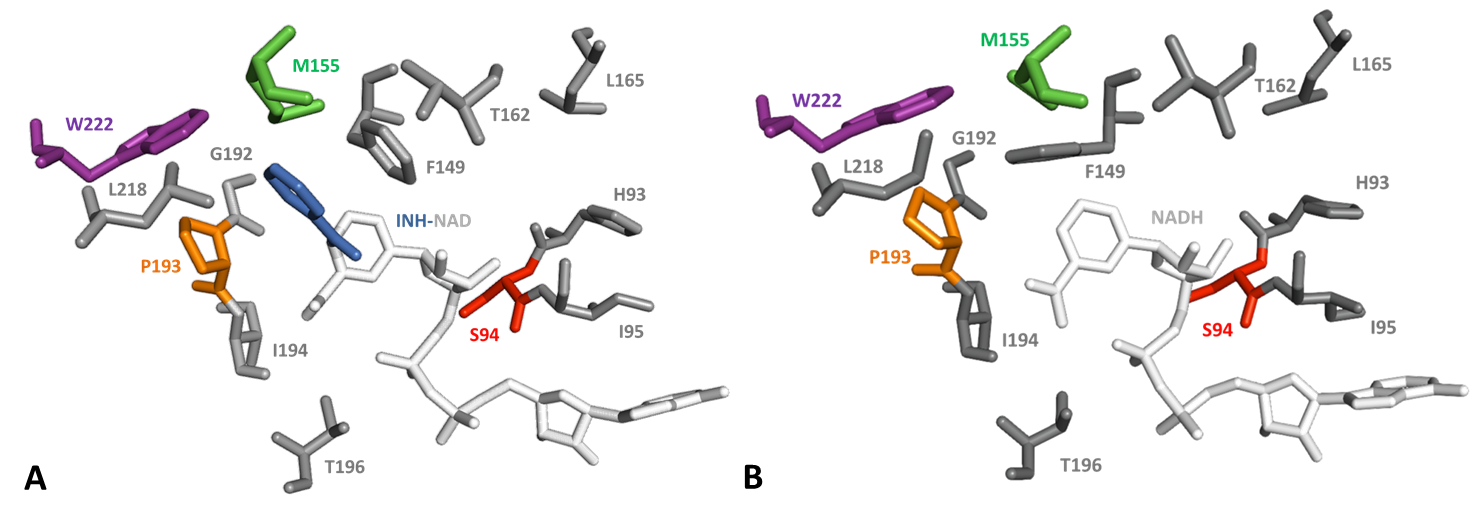


**Fig S1. A.** PDB Entry 1ZID of InhA showing the locations of the residues mutagenised in this work in colour and the INH-NAD adduct indicated in blue (isonicotinoyl moiety) and white (NAD moiety). Residues in close proximity to the adduct but not mutagenised in this study are shown in grey. **Fig S1. B.** depicts PDB entry 2AQ8 in which NADH is bound to InhA using the same colour coding scheme as in **A**.
